# Poly (ADP‐ribose) polymerase inhibition protects against myocardial ischaemia/reperfusion injury via suppressing mitophagy

**DOI:** 10.1111/jcmm.14573

**Published:** 2019-08-05

**Authors:** Shengchuan Cao, Yiying Sun, Wenjun Wang, Bailu Wang, Qun Zhang, Chang Pan, Qiuhuan Yuan, Feng Xu, Shujian Wei, Yuguo Chen

**Affiliations:** ^1^ Department of Emergency and Chest Pain Center Qilu Hospital of Shandong University Jinan China; ^2^ Clinical Research Center for Emergency and Critical Care Medicine of Shandong Province, Institute of Emergency and Critical Care Medicine of Shandong University Qilu Hospital of Shandong University Jinan China; ^3^ Key Laboratory of Emergency and Critical Care Medicine of Shandong Province, Key Laboratory of Cardiopulmonary‐Cerebral Resuscitation Research of Shandong Province Qilu Hospital of Shandong University Jinan China; ^4^ The Key Laboratory of Cardiovascular Remodeling and Function Research, Chinese Ministry of Education, Chinese Ministry of Health and Chinese Academy of Medical Sciences, The State and Shandong Province Joint Key Laboratory of Translational Cardiovascular Medicine Qilu Hospital of Shandong University Jinan China; ^5^ Clinical Trial Center Qilu Hospital of Shandong University Jinan China

**Keywords:** cell apoptosis, mitochondrial membrane potential, mitophagy, poly(ADP‐ribose) polymerase, poly‐ADP‐ribosylation

## Abstract

Myocardial ischaemia/reperfusion (I/R) injury attenuates the beneficial effects of reperfusion therapy. Poly(ADP‐ribose) polymerase (PARP) is overactivated during myocardial I/R injury. Mitophagy plays a critical role in the development of myocardial I/R injury. However, the effect of PARP activation on mitophagy in cardiomyocytes is unknown. In this study, we found that I/R induced PARP activation and mitophagy in mouse hearts. Poly(ADP‐ribose) polymerase inhibition reduced the infarct size and suppressed mitophagy after myocardial I/R injury. In vitro, hypoxia/reoxygenation (H/R) activated PARP, promoted mitophagy and induced cell apoptosis in cardiomyocytes. Poly(ADP‐ribose) polymerase inhibition suppressed H/R‐induced mitophagy and cell apoptosis. Parkin knockdown with lentivirus vectors inhibited mitophagy and prevented cell apoptosis in H/R‐treated cells. Poly(ADP‐ribose) polymerase inhibition prevented the loss of the mitochondrial membrane potential (ΔΨm). Cyclosporin A maintained ΔΨm and suppressed mitophagy but FCCP reduced the effect of PARP inhibition on ΔΨm and promoted mitophagy, indicating the critical role of ΔΨm in H/R‐induced mitophagy. Furthermore, reactive oxygen species (ROS) and poly(ADP‐ribosylation) of CypD and TSPO might contribute to the regulation of ΔΨm by PARP. Our findings thus suggest that PARP inhibition protects against I/R‐induced cell apoptosis by suppressing excessive mitophagy via the ΔΨm/Parkin pathway.

## 1 INTRODUCTION

Reperfusion therapy is the most effective treatment for acute myocardial infarction, reduces ischaemic injury and limits the infarct size. However, reperfusion can independently induce myocardial injury including cardiomyocyte death, leading to expansion on the scope of myocardial infarction.^1^ Myocardial ischaemia/reperfusion (I//R) injury may account for up to 50% of the infarct size.^1^ Despite great effort to optimize reperfusion conditions, the means of reducing I/R injury–associated cell death are very limited.^2^

Several critical factors mediate the detrimental effects of I/R‐induced injury, including oxidative stress, intracellular Ca^2+^ overload, inflammation and mitochondrial permeability transition pore (MPTP) opening.^1^, ^2^ The opening of the MPTP is related to poly(ADP‐ribose) polymerase (PARP) activation, and PARP inhibition prevents myocardial I/R injury.^3^, ^4^ PARP is a highly conserved DNA‐binding nuclear enzyme family that can be activated by DNA damage. Poly(ADP‐ribose) polymerase plays an important role in the regulation of multiple physiological cellular functions including DNA repair, transcription, cell cycle, cell death and genomic integrity.^5^ The activation of PARP initiates an energy‐consuming cycle by transferring ADP‐ribose units from nicotinamide adenine dinucleotide (NAD) to form long branches of ADP‐ribose polymers (PAR) on glutamic acid residues of a number of target proteins.^6^

Mitochondria generate most of the energy for the heart via oxidative phosphorylation. To maintain a healthy and functional mitochondrial network, dysfunctional or damaged mitochondria are eliminated via a process known as mitochondrial autophagy or mitophagy, which is triggered by starvation, hypoxia and reactive oxygen species (ROS).^7^, ^8^ Mitophagy has been classified into canonical and non‐canonical pathways in the heart.^8^ The Parkin‐dependent pathway is the main form of canonical mitophagy.^9^

Poly(ADP‐ribose) polymerase activation has been shown to prevent mitophagy in xeroderma pigmentosum group A‐deficient cells, and PARP inhibition by the inhibitor olaparib induces mitophagy in BRCA1 and BRCA2 mutant breast cancer cells.^10^, ^11^ However, whether PARP activation regulates mitophagy in I/R‐injured cardiomyocytes remains unclear. This study showed that PARP inhibition attenuated I/R‐ or hypoxia/oxygenation (H/R)‐induced mitophagy and cell apoptosis in vivo and in vitro.

## 2 MATERIALS AND METHODS

### 2.1 Reagents and antibodies

The Parkin‐siRNA lentivirus and control lentivirus were constructed by Genechem. 3,4‐dihydro‐5‐[4‐(1‐piperidinyl)butoxy]‐1(2H)‐isoquinoline (DPQ) was from Apexbio (Houston, USA). Cyclosporin A and carbonyl cyanide‐4‐(trifluoromethoxy)phenylhydrazone (FCCP) was from Selleck. N‐acetyl cysteine was from Beyotime (S0077). Anti‐PAR, anti‐Parkin, anti‐COX IV, anti‐cyclophilin D (CypD) and anti‐translocator protein (TSPO) was from Abcam. Anti‐poly(ADP‐ribose) polymerase 1 (PARP‐1) was from Proteintech. Secondary antibodies for Western blotting were from Cell Signaling Technology.

### 2.2 Mice experiments

Acute myocardial I/R model was performed on adult male C57BL/6 mice (10‐12 weeks) by ligating the left anterior descending artery (LAD). Mice were anaesthetized with isoflurane and ventilated using a Rodent Anesthesia Machine. After taped to a heating pad in the supine position, mice chest was opened at the third intercostal space and the heart was exposed by squeezing. A 6‐0 silk suture was passed under the LAD 1‐2 mm from the tip of the left atrium. Left anterior descending artery was ligated with a slipknot. The occlusion was maintained for 30 minutes and then the knot was released to reperfuse the heart for 120 minutes.^12^ To determine the myocardial infarct size, hearts were collected and sectioned into 2‐3 mm slices. The slices were incubated in 1% 2, 3, 5‐triphenyltetrazolium chloride (TTC) solution at 37°C for 20 minutes. Images were photographed and analysed using Image J software (NIH). Cardiac function (left ventricular ejection fraction, LVEF) was assessed in mice by echocardiography (VEVO 2100, VisualSonics) at the end of the 24‐h reperfusion. For PARP inhibition, DPQ (5 mg/kg) was intraperitoneally injected 30 minutes before surgery. Mice were fed rodent chow ad libitum and housed with a 12‐hour light/dark cycle. All aspects of mice care and experimentation were performed in accordance with the Guide for the Care and Use of Laboratory Animals and approved by the Institutional Animal Care and Use Committee of Shandong University.

### 2.3 Cell culture

H9C2 cells or AC16 cells (Shanghai Super Biological Technology) were cultured in Dulbecco's modified Eagle's medium (DMEM) (Gibco) supplemented with 10% foetal bovine serum (FBS), 100 U/ml penicillin and streptomycin. Cells were maintained in a humidified incubator with 95% air/5% CO_2_ at 37°C. For stimulated I/R in vitro, cells were first washed with PBS, incubated with DMEM without FBS and placed in a hypoxia chamber (1% O_2_/5% CO_2_/94% N_2_) for 10 hours, and then cells were returned to normal atmosphere (95% air/5% CO_2_) and incubated with standard culture medium for 2 hours.

### 2.4 Western blot analysis

Protein was extracted from cardiac tissue and H9C2 cells. Equal amounts of protein samples were separated by SDS‐PAGE and electrotransferred to PVDF membranes (Millipore), which were blocked with 5% non‐fat milk for 2 hours at room temperature. Blots were washed with TBS‐T 3 times for 10 minutes and incubated with the primary antibodies overnight at 4°C. Then, blots were incubated with corresponding horseradish peroxidase‐conjugated secondary antibodies for 2 hours at room temperature. Finally, blots were visualized with enhanced chemiluminescence substrate and analysed with Image J software.

### 2.5 Cell apoptosis assay

Apoptotic cells were determined by terminal deoxynucleotidyl transferase‐mediated dUTP‐biotin nick end labelling (TUNEL) assay with Apoptosis Assay Kit (Roche) or were detected by flow cytometry with PI/Annexin V Apoptosis Detection kits (BD Biosciences).

### 2.6 Electron microscopy

Sections of myocardium or cell on coverslips were fixed in 2.5% glutaraldehyde overnight. After rinsing in 0.1 mmol/L cacodylate buffer with 1% tannic acid, the samples were immersed in 1% osmium tetroxide in 0.1 mmol/L cacodylate buffer for 1 hour. After being rinsed again, the samples were dehydrated with alcohol and embedded in Epon 812. Then, the samples were examined using a transmission electron microscope.

### 2.7 Mitochondria isolation

Mitochondria from cultured H9C2 cells were isolated with the Mitochondria Isolation Kit (Thermo Scientific) referred to the manufacturer's instruction.

### 2.8 Measurement of mitochondrial membrane potential (ΔΨm)

ΔΨm was determined using a commercial assay kit by incubation with JC‐1(Beyotime) in serum‐free medium for 20 minutes at 37°C. Then cells were washed with JC‐1 staining buffer and imaged under a fluorescence microscope (Olympus). Normal mitochondria produce red fluorescence, and depolarized or inactive mitochondria produce green fluorescence. ΔΨm was calculated by the red/green fluorescence ratio.

### 2.9 ROS production

Reactive oxygen species were determined with the Reactive Oxygen Species Kit (Beyotime). According to the instruction, cells were incubated with DCFH‐DA for 20 minutes at 37°C. The ROS level was examined in a Thermo Fisher Varioskan Flash spectral scanning multimode reader.

### 2.10 Immunoprecipitation

Poly(ADP‐ribosylation) of CypD and TSPO was detected using immunoprecipitation method.^13^ Lysates of H9C2 cells were prepared with NP‐40 Lysis Buffer (Bosterbio). For the immunoprecipitation studies, 800 μg proteins were incubated with 10 μg PAR antibody followed by incubation with 60 μl protein A/G agarose (Santa Cruz Biotechnology) and the pellets were washed four times. Then, beads were added 40 μl 2 × SDS‐PAGE loading buffer and boiled for 5 minutes to elute the immunocomplexes. Supernatants were subjected to SDS‐PAGE and analysed for CypD and TSPO. Similar procedures were performed to determine the protein‐protein interaction between PARP‐1 and CypD or TSPO.

### 2.11 NAD and ATP measurements

Nicotinamide adenine dinucleotide was measured with a NAD/NADH Assay Kit (Beyotime), and ATP was detected with an ATP Assay Kit (Beyotime). The level of NAD and ATP was normalized to total protein content which was determined by the bicinchoninic acid method.

### 2.12 Statistical analysis

All data were presented as the mean ± SEM and analysed by either one‐way ANOVA or a two‐tailed Student's *t* test. The null hypothesis was rejected at *P* < .05.

## 3 RESULTS

### 3.1 PARP inhibition reduces infarct size and suppresses mitophagy in I/R‐injured hearts

**Figure 1:**
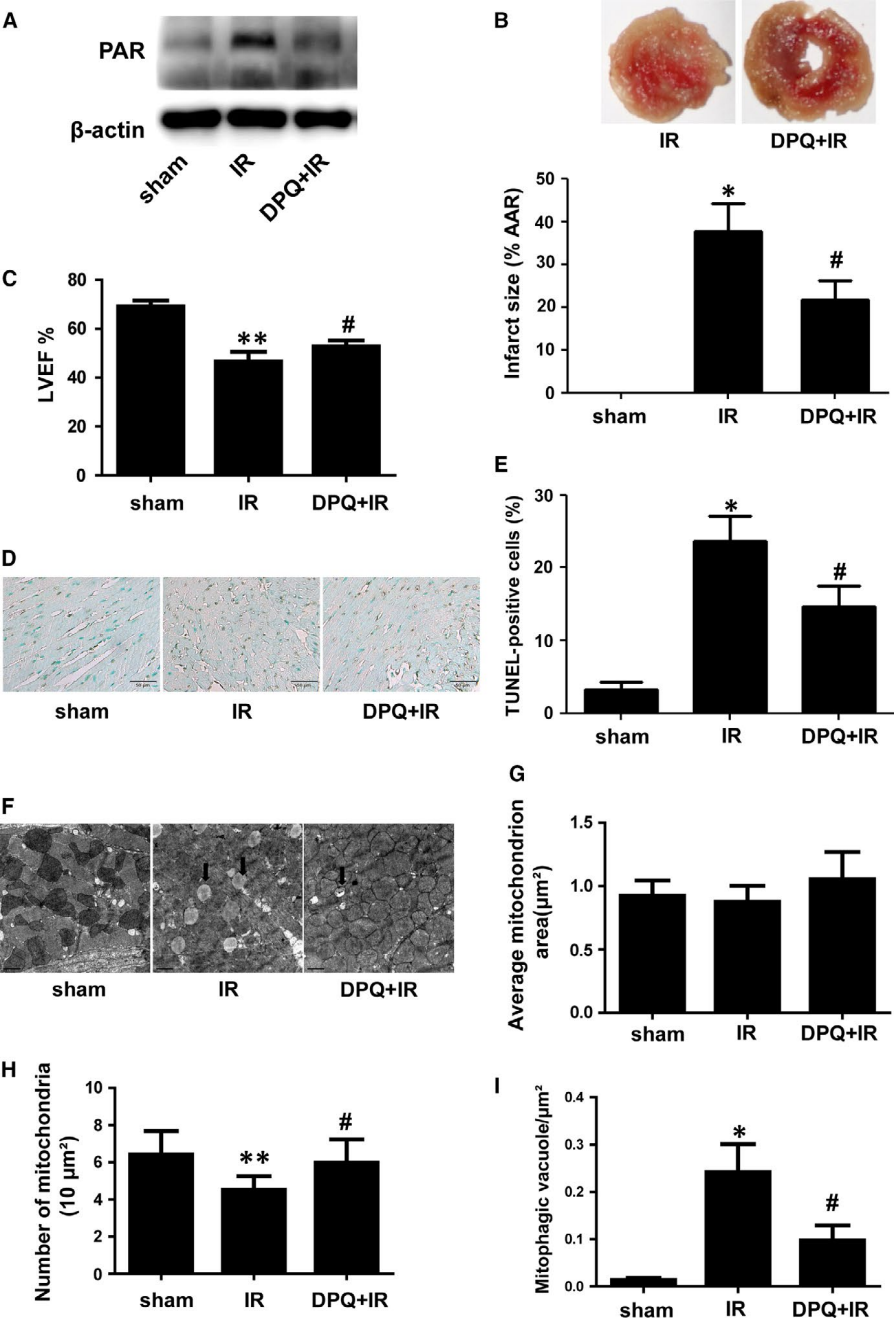
I/R‐induced myocardial injury and mitophagy were attenuated by PARP inhibition. A, Protein expression of PAR indicated by Western blotting. (n = 5). B, TTC staining of heart sections and quantification analysis of the infarct size. (n = 6). C, Cardiac function was determined via echocardiography and indicated by LVEF at the end of the 24‐hour reperfusion. (n = 6). D and E, TUNEL staining showing apoptotic cells in the area at risk after I/R and quantification analysis. (n = 6). F, Electron microscopy showing the structure of mitochondria in the area at risk. (n = 5). Scale bar: 1 μm. G, The average area of individual mitochondria. H, The number of mitochondria per 10 μm^2^ of the cytoplasm. I, Autophagic vacuoles (arrowheads) normalized by the cytoplasmic surface area (per μm^2^). *P < .01 vs sham group, **P < .05 vs sham group, ^#^ *P* < .05 vs I/R group

The role of PARP inhibition in myocardial I/R injury was investigated using the LAD ligation model. I/R‐induced PARP activation and the inhibitor, DPQ, effectively inhibited PARP activity, as indicated by PAR expression (Figure 1A). As expected, PARP inhibition limited the increase of the infarct size compared with the I/R group (Figure 1B). Moreover, PARP inhibition improved the cardiac function after IR injury, as indicated by LVEF (Figure 1C). Terminal deoxynucleotidyl transferase‐mediated dUTP‐biotin nick end labelling staining indicated there were fewer apoptotic cells in the area at risk in the DPQ + I/R group compared with the number of apoptotic cells in the area at risk in the I/R group (Figure 1D,E).

To determine whether I/R induces mitophagy in cardiomyocytes and determine the role of PARP inhibition in mitophagy, we observed and analysed mitochondria in the area at risk using transmission electron microscopy (Figure 1F). No differences were found in regard to the average size of mitochondria among the three groups (Figure 1G). Ischemia/reperfusion injury decreased the number of mitochondria and promoted the formation of autophagic vacuoles containing mitochondria (entire or almost degraded) in the area at risk (Figure 1H,I). However, PARP inhibition prevented changes in the number of mitochondria and the formation of autophagic vacuoles induced by I/R injury (Figure 1H,I).

### 3.2 PARP inhibition prevents cell apoptosis and mitophagy in H/R‐treated cardiomyocytes

**Figure 2:**
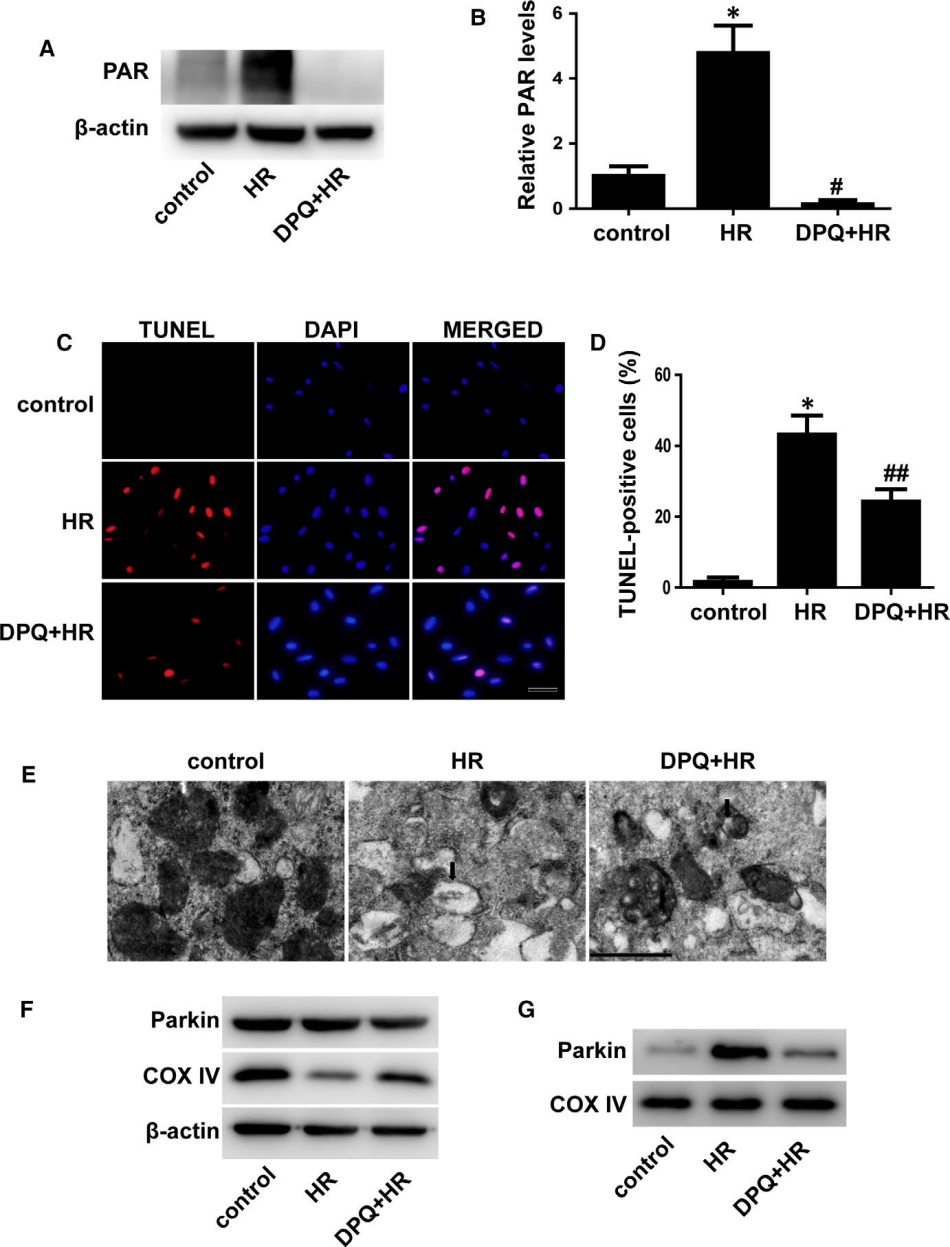
PARP inhibition suppressed H/R‐induced cell apoptosis and mitophagy. A and B, Representative blots of PAR and quantification analysis. (n = 3). C and D, Apoptotic cells were indicated by TUNEL staining (red), and the percentages of apoptotic cells were calculated. (n = 5). Scale bar: 20 μm. E, Autophagic vacuoles were visualized by an electron microscope. Scale bar: 0.5 μm. F, Representative Western blots of Parkin and COX IV from whole cells. (n = 3). G, Proteins from isolated mitochondria were immunoblotted for Parkin and COX IV. (n = 3). *P < .01 vs control, ^#^ *P* < .01 vs H/R group, ^##^ *P* < .05 vs H/R group

To test the effects of PARP inhibition on cell apoptosis and mitophagy after I/R injury in vitro, we subjected H9C2 cells to hypoxia/reoxygenation (H/R) treatment. Figure 2A,B indicated that DPQ effectively inhibited H/R‐induced PARP activation. Consistent with the in vivo results, PARP inhibition protected against cell apoptosis in H/R‐treated H9C2 cells (Figure 2C,D). The role of PARP inhibition in cell death was confirmed by flow cytometry assays (Figure S1). To determine the role of PARP inhibition on mitophagy, we first used an electron microscope to observe mitophagy and found that PARP inhibition decreased autophagic vacuoles after H/R injury (Figure 2E). Mitophagy causes proteolysis of mitochondrial proteins.^14^ Compared to the control, COX IV was decreased in H/R‐treated cells (Figure 2F). Poly(ADP‐ribose) polymerase inhibition prevented the decrease of COX IV after H/R injury (Figure 2F). Although the expression of Parkin in whole cells was not changed, more Parkin was found in proteins from isolated mitochondria after H/R and this trend was prevented by PARP inhibition (Figure 2F,G). Furthermore, AC16 cells were used to confirm the effect of PARP inhibition on cell apoptosis and mitophagy after H/R injury. Consistent with the results in H9C2 cells, we found that DPQ prevented cell apoptosis and mitophagy in H/R‐treated AC16 cells (Figure S2).

### 3.3 Knockdown of Parkin prevents mitophagy and cell apoptosis in H/R‐treated cardiomyocytes

**Figure 3:**
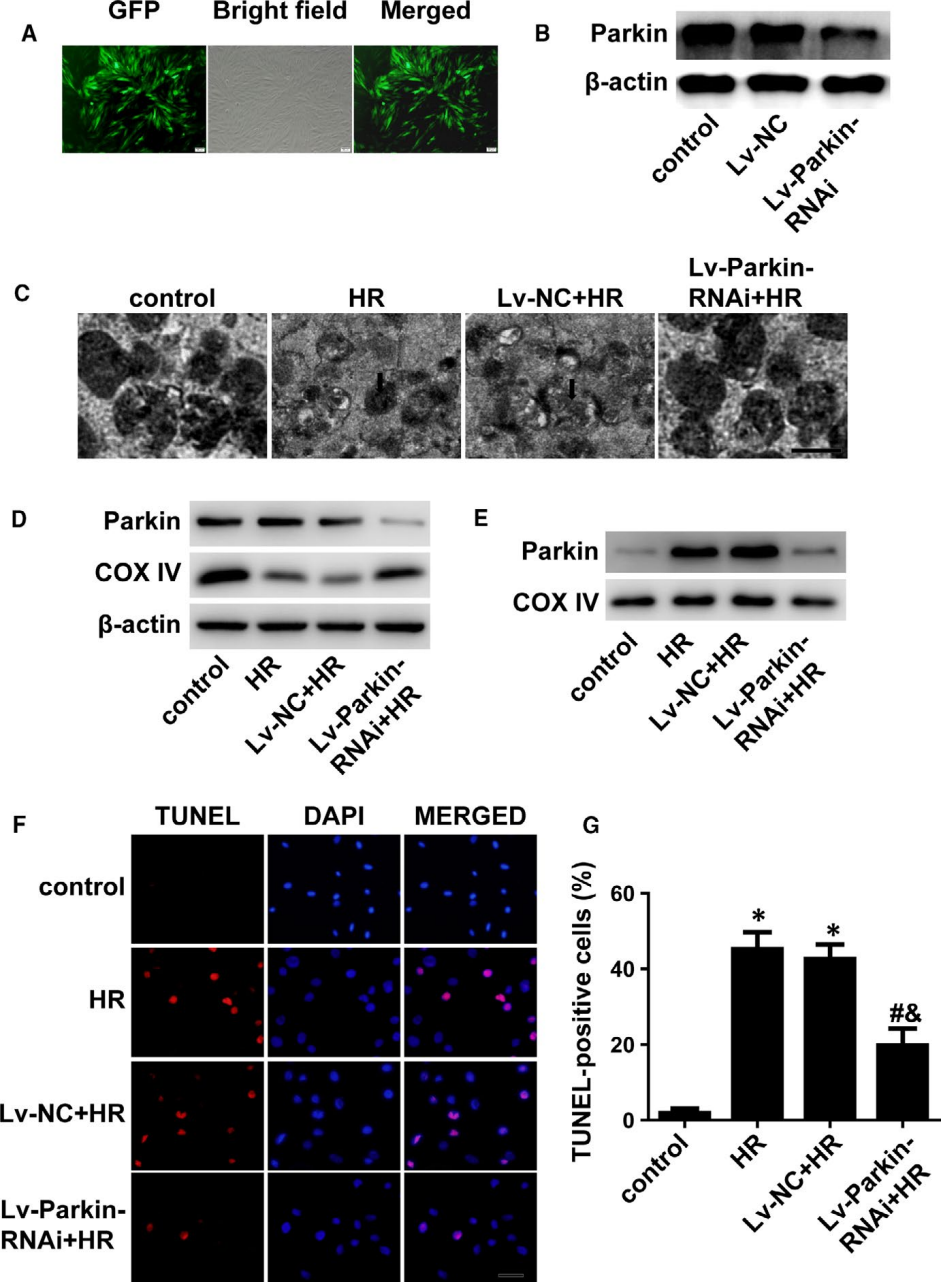
Mitophagy promoted cell apoptosis. A, H9C2 cells were transfected with lentiviruses and photographed with a fluorescence microscope. B, Western blotting indicating the efficacy of Lv‐Parkin‐siRNA. C, Autophagic vacuoles were observed by an electron microscope. Scale bar: 0.5 μm. D, Expression of Parkin and COX IV via immunoblotting of whole cells. (n = 3). E, Proteins from isolated mitochondria were immunoblotted for Parkin and COX IV. (n = 3). F and G, TUNEL staining showing apoptotic cells and quantification analysis. (n = 5). Scale bar: 20 μm. *P < .01 vs control, ^#^ *P* < .05 vs H/R group, ^&^ *P* < .05 vs Lv‐NC + H/R group

To determine the relationship between mitophagy and cell apoptosis in H/R‐treated H9C2 cells, Parkin was knocked down with lentivirus‐RNAi in H9C2 cells. Figure 3A,B shows that Parkin was effectively reduced after transinfection of Lenti‐Parkin‐RNAi into H9C2 cells. Using an electron microscope, we found few autophagic vacuoles after knockdown of Parkin (Figure 3C). Knockdown of Parkin did not change the level of COX IV under normal conditions (Figure S3), but Parkin deficiency inhibited the decrease of COX IV and reduced the level of Parkin in mitochondria after H/R injury (Figure 3D,E). Terminal deoxynucleotidyl transferase‐mediated dUTP‐biotin nick end labelling staining demonstrated that cell apoptosis was inhibited in Parkin‐deficient H9C2 cells (Figure 3F,G). Therefore, excessive mitophagy plays an important role in H/R‐induced cell apoptosis.

### 3.4 PARP inhibition prevents H/R‐induced mitophagy by regulating the mitochondrial membrane potential (ΔΨm)

**Figure 4:**
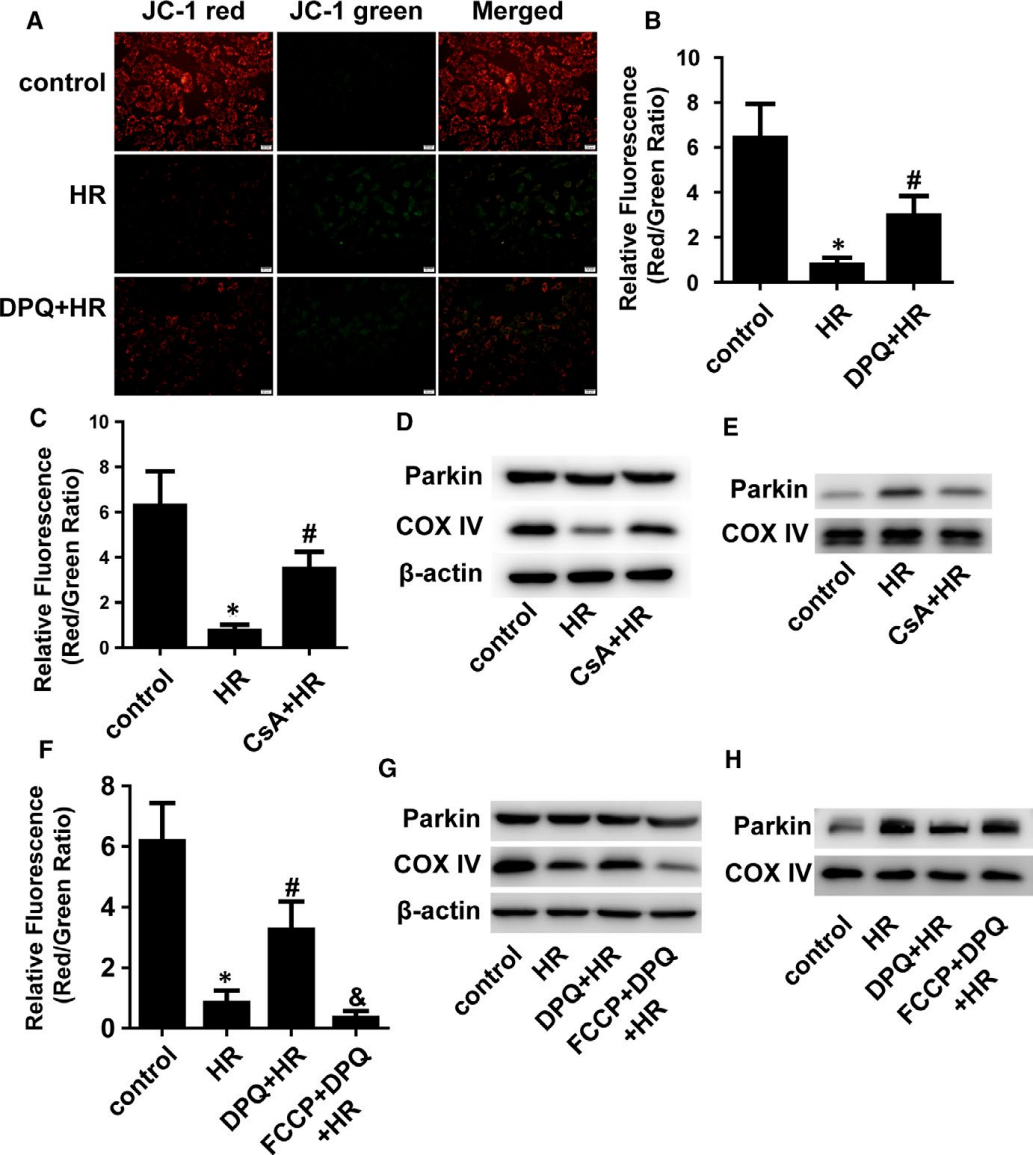
ΔΨm mediated the effect of PARP on mitophagy. A, ΔΨm was determined by JC‐1 staining. Scale bar: 20 μm. B, C, and F, The relative ΔΨm was quantitatively analysed. (n = 5). D and G, Total proteins were immunoblotted for Parkin and COX IV. (n = 3). E and H, Proteins from isolated mitochondria were immunoblotted for Parkin and COX IV. (n = 3). *P < .01 vs control, ^#^ *P* < .05 vs H/R group, ^&^ *P* < .01 vs DPQ + H/R group

Opening of the MPTP causes the loss of ΔΨm, which triggers PINK1/Parkin‐mediated mitophagy.^15^, ^16^ ΔΨm was obviously reduced by H/R injury, and mitochondrial membrane depolarization was restored by PARP inhibition (Figure 4A,B). To ensure the critical role of ΔΨm in mitophagy in H/R‐injured H9C2 cells, we pre‐incubated H9C2 cells with cyclosporin A, which is a potent inhibitor of the MPTP. Figure 4C shows that cyclosporin A prevented the disruptive effect of H/R on ΔΨm. Similar to PARP inhibition, cyclosporin A prevented mitophagy in H/R‐injured H9C2 cells (Figure 4D,E). FCCP is an oxidative phosphorylation uncoupler that depolarizes the mitochondrial membrane.^17^ It was found that the effect of PARP inhibition on ΔΨm and mitophagy was eliminated by FCCP (Figure 4F‐H). Collectively, these results indicate that ΔΨm plays a critical role in the regulation of H/R‐induced mitophagy by PARP.

### 3.5 ROS contributes to the PARP‐mediated decline in ΔΨm

**Figure 5:**
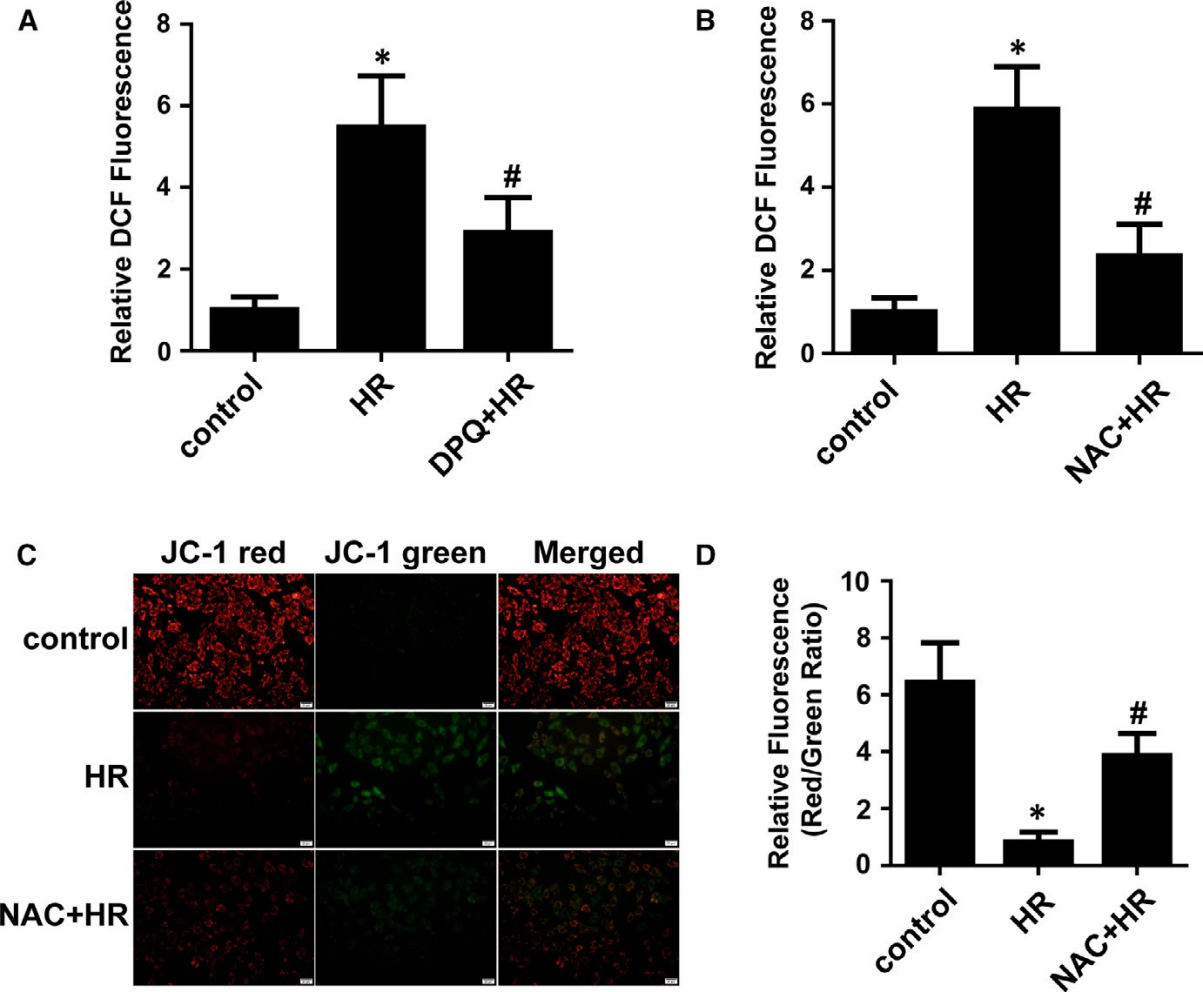
ROS participated in the disruptive effect of PARP on ΔΨm. A and B, The levels of ROS were determined with DCF, and the relative mean DCF fluorescence was expressed compared with the control. (n = 5). C and D, ΔΨm was determined by JC‐1 and quantification analysis. (n = 5). *P < .01 vs control, ^#^ *P* < .05 vs H/R group

Excessive ROS trigger the opening of the MPTP and activate PINK1/Parkin‐mediated mitophagy.^18^ To investigate whether ROS play a role in PARP‐mediated changes in ΔΨm, we measured ROS production in H/R‐treated H9C2 cells. The production of ROS was significantly increased in H/R‐treated H9C2 cells compared with that in control cells, and PARP inhibition partially prevented the increase in ROS production after H/R (Figure 5A). The reductant NAC reduced the production of ROS (Figure 5B). More importantly, we found that NAC prevented the H/R‐induced decline in ΔΨm (Figure 5C,D). Thus, ROS have an effect, at least partially on the changes in ΔΨm mediated by PARP after H/R.

### 3.6 Poly‐ADP‐ribosylation of CypD and TSPO may also contribute to the PARP‐mediated decline in ΔΨm

**Figure 6:**
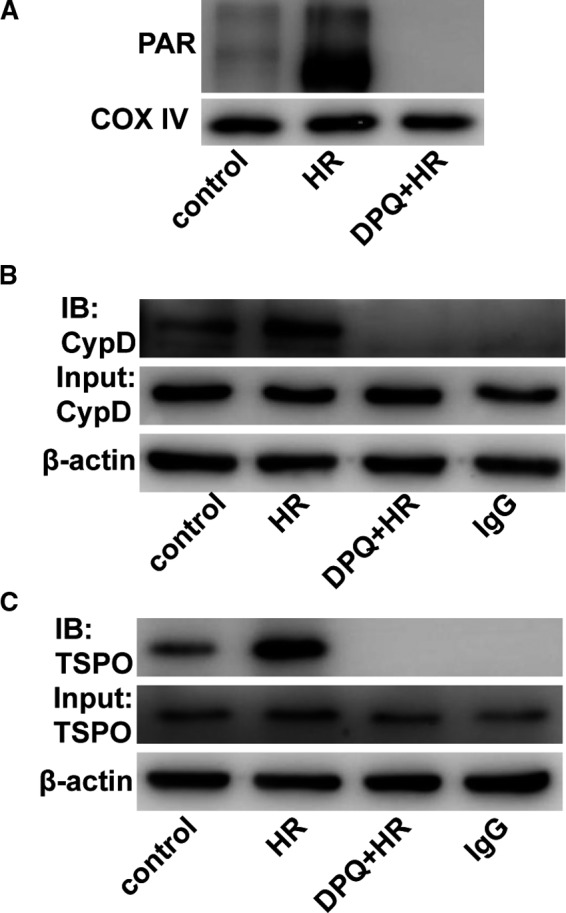
Mitochondrial CypD and TSPO were modified by poly(ADP‐ribosylation). A, Proteins from isolated mitochondria were immunoblotted for Parkin and COX IV. (n = 3). B, Immunoprecipitation using a PAR antibody showed that CypD was modified by poly(ADP‐ribosylation). (n = 5). C, Immunoprecipitation using a PAR antibody showed that TSPO was modified by poly(ADP‐ribosylation). (n = 5)

Poly(ADP‐ribose) polymerase activation induces poly‐ADP‐ribosylation of mitochondrial proteins after I/R.^19^ Immunoblotting indicated that more PAR was found in proteins of isolated mitochondria after H/R injury (Figure 6A). CypD and TSPO have been identified as the MPTP components and contribute to the regulation of the opening of the MPTP.^20^, ^21^ We tried to determine whether CypD and TSPO could be poly‐ADP‐ribosylated in H/R‐treated H9C2 cells. Via immunoprecipitation, we found that both CypD and TSPO could be modified by poly‐ADP‐ribosylation and that PARP inhibition effectively prevented this modification (Figure 6B,C). However, we did not find a direct interaction between PARP‐1 and CypD or TSPO (Figure S4). These data demonstrate that PARP might regulate the opening of the MPTP by directly modifying CypD and TSPO by poly‐ADP‐ribosylation.

## 4 DISCUSSION

In the present study, we found that PARP inhibition prevents I/R injury–induced mitophagy and cell apoptosis in cardiomyocytes. Mitophagy inhibition due to knockdown of Parkin decreases H/R‐induced cell apoptosis, demonstrating that excessive mitophagy reduces cell survival. The loss of ΔΨm is reversed by PARP inhibition, and changes in ΔΨm play a critical role in PARP‐mediated mitophagy. Furthermore, ROS and modification of CypD and TSPO by poly‐ADP‐ribosylation might contribute to the regulation of ΔΨm.

In contrast to PARP's protective role in the case of a low level of DNA damage, PARP activation acts to promote cell death in the presence of extensive DNA damage caused by oxidative stress.^6^ PARP serves as a molecular switch between apoptosis and necrosis. Overactivation of PARP depletes intracellular NAD and ATP which results in a cellular energy crisis and irreversible cytotoxicity, leading to cell necrosis.^6^ Moderate activation of PARP leads to a unique form of cell apoptosis mediated via accumulation of cytosolic PAR and nuclear translocation of apoptosis‐inducing factor (AIF) from mitochondria.^3^ PARP activation also induces mitochondrial membrane depolarization and MPTP inhibition with cyclosporin A prevents the effect of PARP activation on ΔΨm indicating mitochondrial function might play an important role in PARP‐mediated cell death.^22^ Consistent with previous studies,^23^, ^24^ we also found that PARP inhibition prevented I/R‐induced cell apoptosis in vivo and in vitro.

Mitophagy facilitates the normal turnover of mitochondria and becomes more important during exposure to stress including I/R injury. Although the role of mitophagy in myocardial I/R injury has been discussed in several studies, it needs to be further explored because of conflicting conclusions.^25^, ^26^, ^27^ Under acute myocardial I/R injury, the MPTP has been shown to remain closed during ischaemia and open during reperfusion in response to oxidative stress, mitochondrial Ca^2+^ overload, ATP depletion and rapid pH recovery.^1^ Opening of the MPTP leads to the disruption of ΔΨm, and loss of ΔΨm induces PINK1/Parkin‐mediated mitophagy. Under the loss of ΔΨm, PINKI steadily accumulates on the surface of the mitochondrial outer membrane in a voltage‐dependent manner and recruits Parkin from the cytosol to mitochondria.^28^ PARP activation causes mitochondrial damage and opening of the MPTP.^22^, ^29^ Therefore, we hypothesized that PARP activation regulates mitophagy in I/R‐injured cardiomyocytes. In this study, mitophagy was found in cardiomyocytes after I/R or H/R injury and PARP inhibition prevented mitophagy. To explore the relationship between mitophagy and cell apoptosis, we suppressed mitophagy via Parkin knockdown and confirmed that excessive mitophagy promotes cell apoptosis during myocardial I/R injury. Moreover, PARP inhibition prevented I/R‐induced depletion of NAD and ATP (Figure S5A,B), whereas Parkin knockdown did not change their levels after I/R injury (Figure S5C,D). Thus, the effect of mitophagy on I/R‐induced cell apoptosis is independent of the level of NAD and ATP.

At the beginning of myocardial reperfusion, a large amount of ROS is produced from a variety of sources including extracellular and intracellular actions.^1^ ROS activate PINKI/Parkin‐mediated mitophagy by promoting the opening of the MPTP and disrupting ΔΨm.^18^ ROS also function as a direct signal to induce mitophagy.^30^ ROS modulate MPTP opening by oxidizing Cys56 and, Cys160 of adenine nucleotide translocator (ANT) and Cys203 of cyclophilin D (CypD), increasing mitochondrial Ca2+ and translocating Bid to jBid.^31^ PARP can be overactivated by oxidative stress, and PARP activation also promotes the generation of ROS.^32^ As expected, we found PARP inhibition was able to reduce the production of ROS after H/R injury. Moreover, the loss of ΔΨm was prevented when cells were pre‐treated with the ROS scavenger NAC. Thus, ROS is likely a downstream signal through which PARP regulates ΔΨm.

Poly(ADP‐ribose) polymerase activation might trigger mitochondrial depolarization and dysfunction by poly‐ADP‐ribosylation of mitochondrial proteins.^3^ Free PAR polymers are released from the nucleus to the cytosol and accumulate around mitochondria after PARP activation.^33^ Intra‐mitochondrial poly‐ADP‐ribosylation also contributes to mitochondrial dysfunction by modifying mitochondrial proteins.^19^ However, it is unclear whether MPTP opening is related to the poly‐ADP‐ribosylation of relative proteins. The protein components of the MPTP still need to be explored. CypD and TSPO are potent components that act as regulators of MPTP opening.^21^, ^34^ Down‐regulation of CypD prevents MPTP opening and protects against the loss of ΔΨm.^35^ Conversely, the loss function of TSPO causes MPTP opening and leads to a reduced ΔΨm.^21^, ^36^ Our results showed that both CypD and TSPO could be poly‐ADP‐ribosylated, but the effects of this modification on the functions of CypD and TSPO were not studied. As reported, poly‐ADP‐ribosylation can serve as a marker for ubiquitin‐proteasomal system (UPS)‐dependent protein degradation.^37^ Thus, we speculate that the function of CypD and TSPO might be changed by poly‐ADP‐ribosylation, which should be investigated in the future.

In conclusion, our study is the first to report that PARP inhibition prevents cell apoptosis by suppressing mitophagy during myocardial I/R injury. ΔΨm is a critical downstream target through which PARP regulates mitophagy. Reactive oxygen species and poly‐ADP‐ribosylation of CypD and TSPO might contribute to the loss of ΔΨm.

## CONFLICT OF INTEREST

The authors confirm that there are no conflicts of interest.

## AUTHOR CONTRIBUTION

Shujian Wei and Yuguo Chen conceived and designed this study. Shujian Wei wrote the manuscript, and Yuguo Chen revised the manuscript. Shengchuan Cao, Yiying Sun, Wenjun Wang and Qiuhuan Yuan performed the experiments. Bailu Wang, Qun Zhang, Chang Pan and Feng Xu analysed the data. All authors read and approved the version of the manuscript to be published.

## Data Availability

The data that support the findings of this study are available from the corresponding author upon reasonable request.
