# Supplementary material for: Poly (ADP‐ribose) polymerase inhibition protects against myocardial ischaemia/reperfusion injury via suppressing mitophagy
Source: J Cell Mol Med. 2019 Aug 5;23(10):6897–906. doi: 10.1111/jcmm.14573 (PMC6787458; doi:10.1111/jcmm.14573)
Supplement: Supplementary file 1 [file JCMM-23-6897-s001.docx]

**Supplemental Figures and Figure Legends**


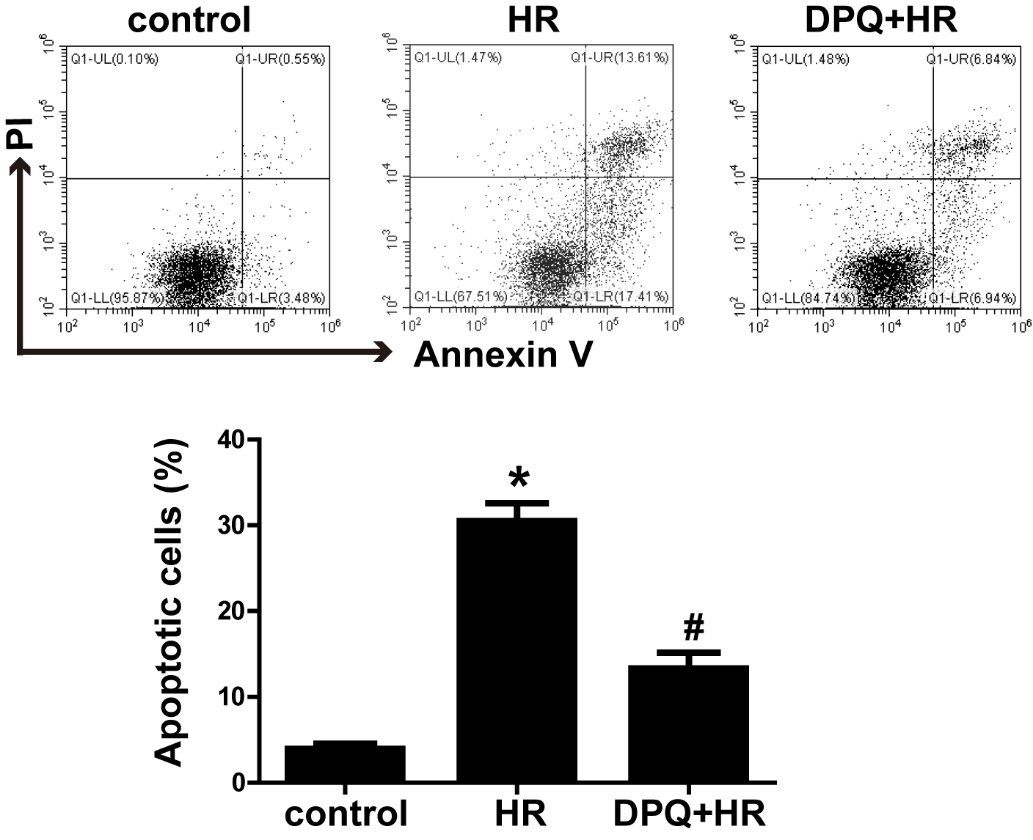


Supplemental Figure 1. Flow cytometry indicated PARP inhibition with DPQ prevented IR-induced cell apoptosis. (n=6). **P*<0.01 vs. control group, ^#^*P*<0.05 vs. H/R group.


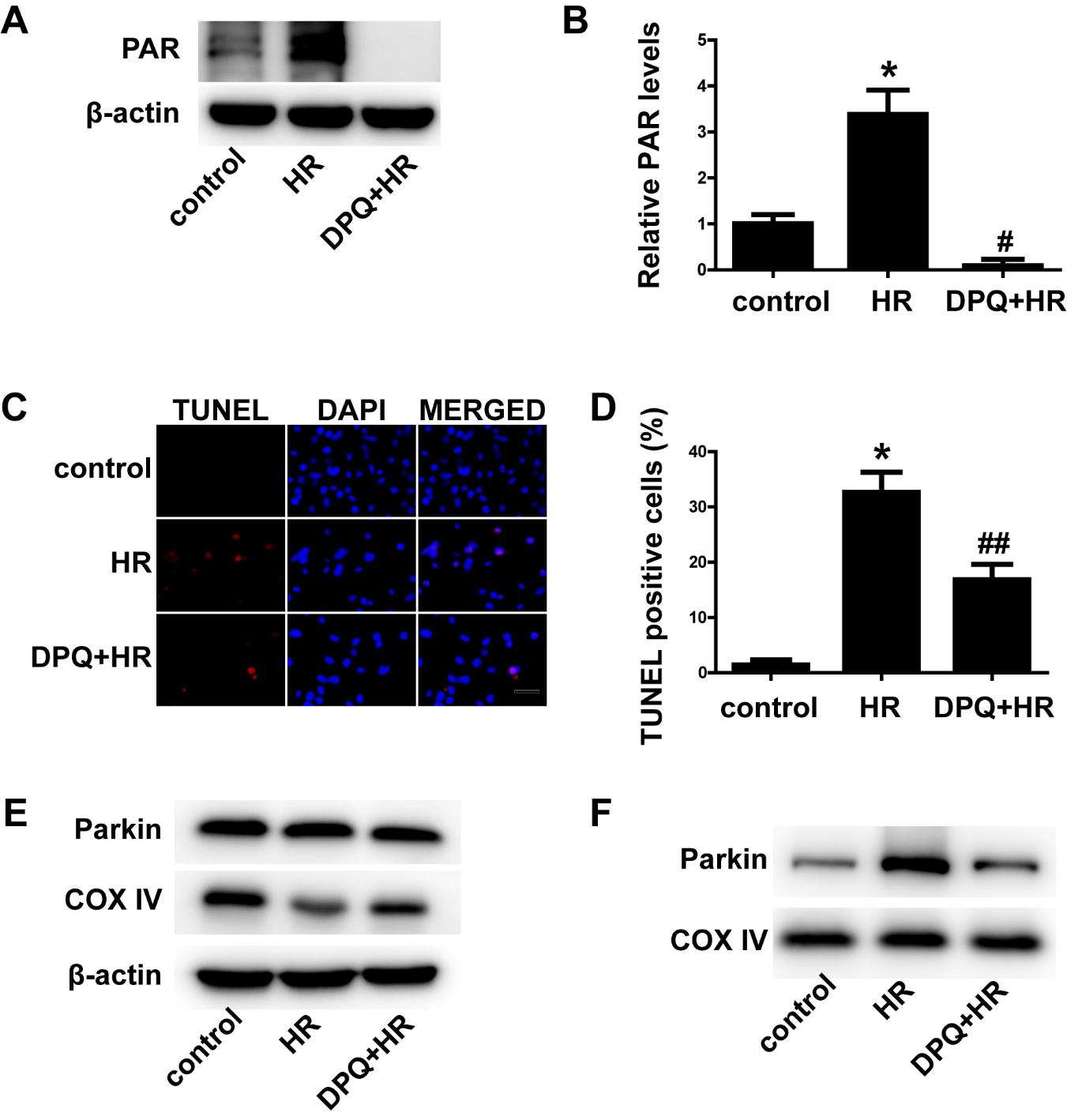


Supplemental Figure 2. DPQ suppressed H/R-induced cell apoptosis and mitophagy in AC16 cells. A and B, Representative blots of PAR and quantification analysis. (n=3). C and D, Apoptotic cells were indicated by TUNEL staining (red) and the percentages of apoptotic cells were calculated. (n=5). Scale bar: 20μm. E, Representative western blots of Parkin and COX IV from whole cells. (n=3). F, Proteins from isolated mitochondria were immunoblotted for Parkin and COX IV. (n=3). **P*<0.01 vs. control, ^#^*P*<0.01 vs. H/R group, ^##^*P*<0.05 vs. H/R group.


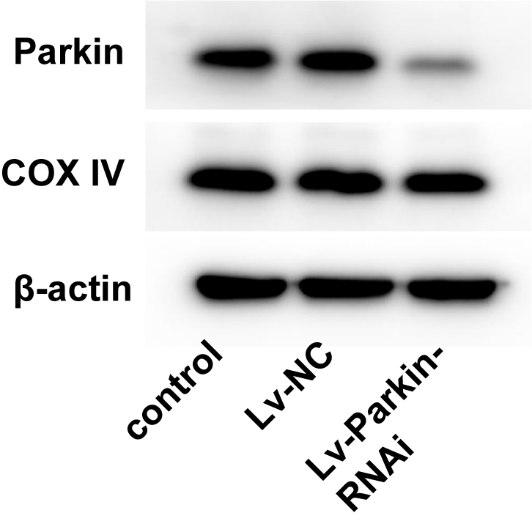


Supplemental Figure 3. Parkin knockdown had no effect on the expression of COX IV under normal condition. (n=5).


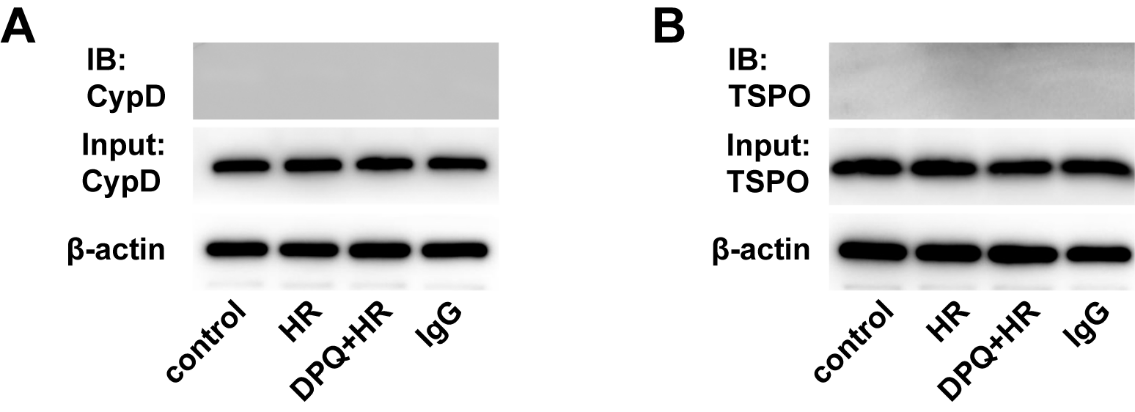


Supplemental Figure 4. Immunoprecipitaion using PARP-1 antibody to indicate the protein-protein interaction between PARP-1 and CypD (A) or TSPO (B). (n=5).


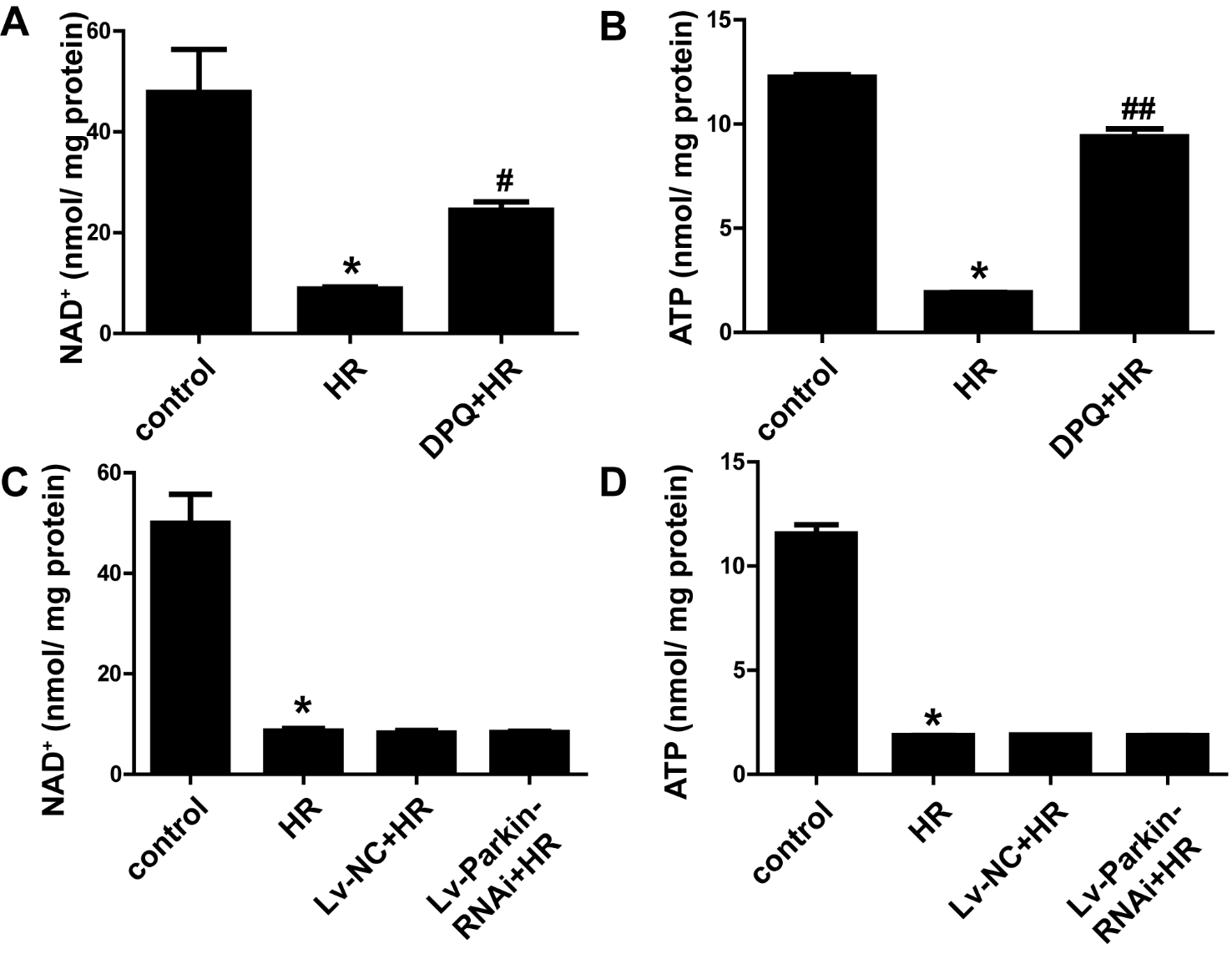


Supplemental Figure 5. PARP inhibition prevented the depletion of NAD (A) and ATP (B) in H/R treated cells. No effect of Parkin knockdown on NAD (C) and ATP (D) was found. **P*<0.01 vs. control group, ^#^*P*<0.05 vs. H/R group, ^##^*P*<0.01 vs. H/R group.
